# An atomistic model of the coronavirus replication-transcription complex as a hexamer assembled around nsp15

**DOI:** 10.1016/j.jbc.2021.101218

**Published:** 2021-09-23

**Authors:** Jason K. Perry, Todd C. Appleby, John P. Bilello, Joy Y. Feng, Uli Schmitz, Elizabeth A. Campbell

**Affiliations:** 1 Gilead Sciences, Inc, Foster City, California, USA; 2 Laboratory of Molecular Biophysics, The Rockefeller University, New York, New York, USA

**Keywords:** SARS-CoV-2, coronavirus, viral replication, viral transcription, protein structure, molecular modeling, structure model, proofreading, nsp15, CTD, C-terminal domain, EndoN, endonuclease, GTase, guanylyltransferase, MD, molecular dynamics, MHV, murine hepatitis virus, MST, microscale thermophoresis, MTase, methyltransferase, N, nucleocapsid, nsp, nonstructural viral protein, NTD, N-terminal domain, NTPase, nucleoside triphosphatase, RTC, replication-transcription complex, TRS, transcription regulatory sequence, TRS-B, body TRS, TRS-L, leader TRS

## Abstract

The SARS-CoV-2 replication-transcription complex is an assembly of nonstructural viral proteins that collectively act to reproduce the viral genome and generate mRNA transcripts. While the structures of the individual proteins involved are known, how they assemble into a functioning superstructure is not. Applying molecular modeling tools, including protein–protein docking, to the available structures of nsp7-nsp16 and the nucleocapsid, we have constructed an atomistic model of how these proteins associate. Our principal finding is that the complex is hexameric, centered on nsp15. The nsp15 hexamer is capped on two faces by trimers of nsp14/nsp16/(nsp10)_2_, which then recruit six nsp12/nsp7/(nsp8)_2_ polymerase subunits to the complex. To this, six subunits of nsp13 are arranged around the superstructure, but not evenly distributed. Polymerase subunits that coordinate dimers of nsp13 are capable of binding the nucleocapsid, which positions the 5′-UTR TRS-L RNA over the polymerase active site, a state distinguishing transcription from replication. Analysis of the viral RNA path through the complex indicates the dsRNA that exits the polymerase passes over the nsp14 exonuclease and nsp15 endonuclease sites before being unwound by a convergence of zinc fingers from nsp10 and nsp14. The template strand is then directed away from the complex, while the nascent strand is directed to the sites responsible for mRNA capping. The model presents a cohesive picture of the multiple functions of the coronavirus replication-transcription complex and addresses fundamental questions related to proofreading, template switching, mRNA capping, and the role of the endonuclease.

Coronaviruses, including the SARS-CoV-2 pathogen responsible for COVID-19, constitute a family of positive sense, single-stranded RNA viruses. After cellular infection, these viruses harness both viral proteins and host machinery to reproduce the viral genome and assemble new viral particles that are released to infect new hosts (1). The replication and transcription of the coronavirus genome are facilitated by a complex of nonstructural viral proteins (nsps) encoded by the *ORF1ab* gene (Fig. 1). The *ORF1a* gene translates a polyprotein, which spans from nsp1 to nsp10. This polyprotein is cleaved into discrete proteins by two encoded proteases, nsp3 and nsp5. After a frameshift, a longer *ORF1ab* polyprotein may be produced, which includes the additional proteins nsp12–nsp16. This set of proteins, in addition to the nucleocapsid (N), which packages the viral RNA, perform the primary functions of RNA synthesis, proofreading, mRNA capping, and strand separation in both full-genome replication mode and subgenomic transcription mode.

**Figure 1:**
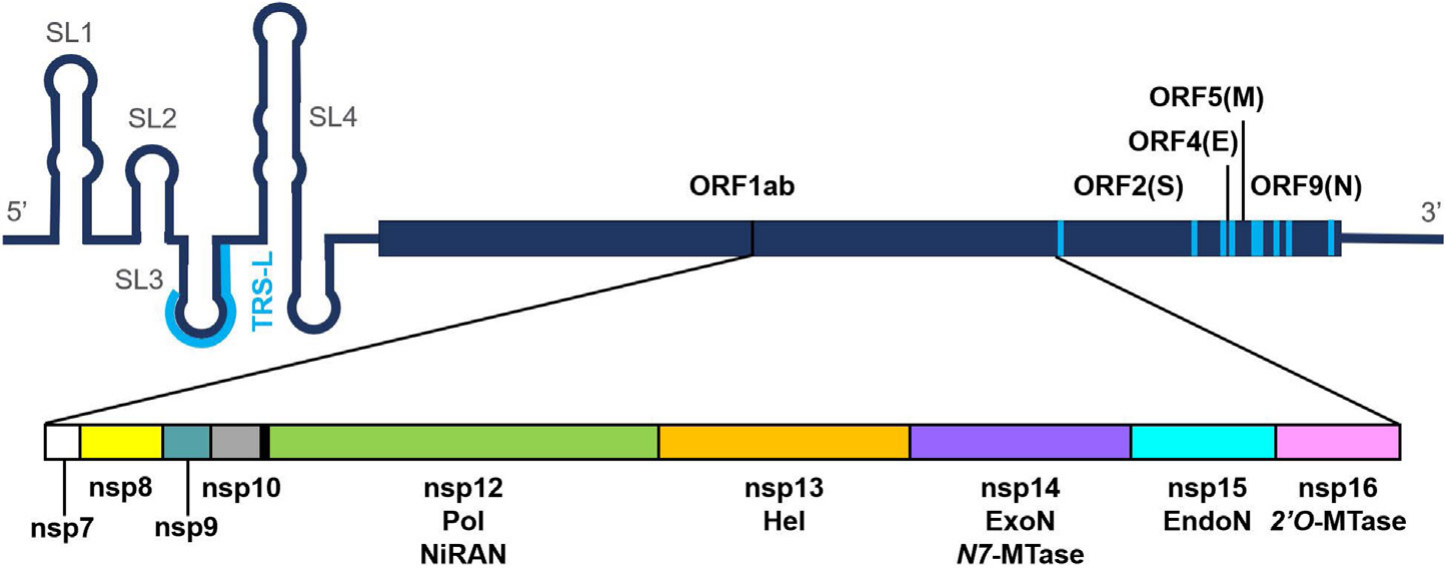
**Organization of the SARS-CoV-2 genome.** The genome is divided into multiple open reading frames (ORFs), with ORF1ab containing the nonstructural proteins (nsps) required for RNA replication (nsp7–nsp16). The structured 5′-UTR leader contains a transcription regulatory sequence (TRS-L), which is repeated throughout the genome, each instance preceding an ORF (indicated in *blue*). During negative-strand synthesis, shorter transcripts may be generated when the template switches from one of the TRS locations in the body of the genome to the TRS-L location in the 5′-UTR, effectively skipping over the regions in between.

The majority of these proteins have been well characterized. Nsp12 contains the polymerase (Pol) active site responsible for RNA synthesis (2). It also contains a second enzymatic site referred to as the NiRAN (nidovirus RdRp-associated nucleotidyltransferase), which is unique to viruses in the *Nidovirales* order (3). The role of this site remains ill-defined, but it has been shown to nucleotidylate proteins such as nsp9 (4), and it has been proposed to play the critical role of guanylyltransferase (GTase) in mRNA capping (5). Nsp14 also has two enzymatic activities (6). Its N-terminal domain (NTD) is an exonuclease (ExoN), which has been shown to be responsible for RNA proofreading during synthesis. Its C-terminal domain (CTD) is an *N7*-methyltransferase (MTase) involved in mRNA capping. Nsp16 is a *2′O*-MTase, also involved in mRNA capping (7). Nsp13 is a helicase (Hel), capable of unwinding RNA, powered by a nucleoside triphosphatase (NTPase) site (8). Nsp15 is an endonuclease (EndoN), which is believed to cleave the 5′ poly-U tail of the intermediate negative-strand (9). And finally, various cofactors such as nsp7, nsp8, nsp9, nsp10, and the nucleocapsid (N) have been shown to be involved in replication and transcription as well (10, 11, 12, 13).

Unlike many other viral families, structures have been determined for all key proteins that are presumed to make up the coronavirus replication-transcription complex (RTC), several of which are shown in Fig. S1. To date, there are X-ray crystal structures of coronavirus nsp13 (14, 15), heterodimeric nsp14/nsp10 (6, 16, 17), heterodimeric nsp16/nsp10 (11, 12, 18, 19, 20, 21), hexameric nsp15 (22, 23, 24, 25, 26, 27), dimeric nsp9 (28, 29, 30), and the N protein NTD bound to both dsRNA and the specific viral RNA oligo known as the transcription regulatory sequence (TRS), which is critical to the unusual template switching process that occurs during transcription (31). Cryo-EM has been especially successful in illuminating the structure of the core polymerase complex, made up of nsp12, nsp7, two subunits of nsp8, and up to two subunits of nsp13 (2, 5, 10, 32, 33, 34, 35, 36). In this complex, nsp7 and the two nsp8 subunits sit atop the nsp12 Pol active site, coordinating to the thumb and fingers domains. The long and flexible N-terminal (N-term) nsp8 helices extend out over the exiting dsRNA when the complex is captured in its replicating state. Coordinated to these nsp8 helices, two subunits of nsp13 sit above the polymerase complex, where one of them has been observed engaging the downstream RNA template overhang.

Yet despite this wealth of structural information, there is no atomistic picture of how the polymerase complex interacts with the remaining proteins to form the complete RTC, leaving major questions of viral RNA processing unanswered. A number of studies have mapped potential protein–protein interactions, with the general conclusion that an extensive interaction network links the cleaved *ORF1ab* proteins together (37, 38, 39, 40). Functionally, it is inferred that the nsp14 ExoN must have some interaction with nsp12 in order to gain access to the 3′ end of the nascent strand to carry out proofreading during RNA synthesis. The dsRNA that emerges from the polymerase must eventually be unwound. To produce transcripts for protein translation, the resulting 5′ end of the nascent positive strand must be directed to the mRNA capping sites: presumably the NiRAN site of nsp12 and the two MTase sites of nsp14 and nsp16. The complicated process of template switching during transcription remains entirely enigmatic but appears to involve N recognizing the specific junctions where the switch occurs: the leader TRS (TRS-L) and body TRS (TRS-B) (Fig. 1) (41).

In this work, we have endeavored through molecular modeling to determine how the many proteins identified above assemble into a functioning SARS-CoV-2 RTC. An observation of how nsp15 interacts with dsRNA led us to hypothesize that it could provide the scaffolding around which the complex could be built. The resulting RTC superstructure is a hexamer, with six subunits each of nsp12, nsp13, nsp14, nsp15, and nsp16. The model demonstrates how RNA makes its way from the Pol active site, across the ExoN and EndoN sites, separates into template and nascent strands, and directs the 5′ end of the nascent strand to the three mRNA capping sites. We have also identified a binding site for the TRS-L bound N protein that has implications for template switching during negative-strand synthesis.

Here we outline how the model was constructed and its overall architecture. We describe the implications on multiple functions associated with the RTC, including proofreading, mRNA capping, template switching, and negative-strand poly-U cleavage. We believe this work presents a cohesive picture of the complicated processes associated with coronavirus genome replication and transcription and offers a roadmap for further exploration.

## Results

### Proteins comprising the RTC

The model was assembled from existing structures of the individual SARS-CoV-2 components (Fig. S1). This included the known SARS-CoV-2 polymerase complex nsp12/nsp7/(nsp8)_2_/(nsp13)_2_/dsRNA (PDB:6XEZ) (32) (Fig. S1*A*), an homology model of nsp14/nsp10 based on a SARS-CoV X-ray structure (PDB:5NFY, chains A/M) (6) (Fig. S1*B*), nsp16/nsp10 (PDB:6WVN) (21) (Fig. S1*C*), hexameric nsp15 (PDB:6X1B) (25) (Fig. S1*D*), dimeric nsp9 (PDB:6W4B) (K. Tan, unpublished results), and the NTD of the N protein bound to the 10-nucleotide (nt) TRS-L oligo (PDB:7ACT) (31) (Fig. S1*E*). Following previous precedent, we refer to the two subunits of nsp8 in the polymerase complex as nsp8.1 and nsp8.2, and the two subunits of nsp13 as nsp13.1 and nsp13.2 (32). Similarly, as there are two sources for nsp10 (associated with nsp14 and nsp16), we refer to them as nsp10.14 and nsp10.16, as necessary for clarity. Finally, as the hexameric nature of the complex turned out to be critical, we adopt a notation based on the nsp15 structure to simplify the discussion. This structure can be viewed as a dimer of trimers (see Fig. S1*D*), presenting two trigonal faces: face A and face B. We refer to the subunits of nsp15 as A1, A2, A3, B1, B2, and B3. This notation will subsequently be used to identify associated subunits of the superstructure.

### Initial protein–protein docking

In protein–protein docking, one protein (or complex of proteins) is treated as the receptor and the other is treated as the ligand. The ligand protein is rotated and translated relative to the receptor protein to optimize the docking score. While the protein–protein interface is subsequently optimized for the top docking poses, major conformational changes to either protein are not sampled. The results of the automated protein–protein docking can be improved with further optimization of the top poses, but ultimately the method works best when the proteins are not highly flexible. As controls, we checked the self-docking of several key proteins, which are the focus of this work. Of these, the interactions of nsp10 with nsp14 (from the 5NFY homology model), nsp10 with nsp16 (from 6WVN), nsp15 monomer with nsp15 pentamer (from 6X1B), and individually nsp7, nsp8.1, and nsp8.2 with the other proteins of the polymerase complex (from 6XEZ) were successfully predicted. The only failure among this control set was the docking of nsp13.1 and nsp13.2 to the polymerase complex. It is unclear if this was simply a sampling issue due to the size of the proteins involved or if the interaction is too weak to identify through this approach.

With this method, we undertook a systematic approach to dock various components of the complex in a binary fashion, with a focus on how additional proteins would interact with the polymerase complex (nsp12/nsp7/(nsp8)_2_/(nsp13)_2_(±RNA)) already established. The outcome of most of these exercises was not particularly fruitful, providing no new insight into the interactions between the polymerase complex and nsp14/nsp10, monomeric nsp15 or nsp16/nsp10. Most of the resulting docking poses for these key proteins suggested more of a preference for interaction with the dsRNA exiting the polymerase than interactions with the proteins of the polymerase complex itself. Similarly, little insight into the interactions within the set of nsp14/nsp10, nsp15, and nsp16/nsp10 could be discerned.

However, two exceptions emerged. The nsp9 dimer, a putative RNA-binding protein, demonstrated a preference for binding to the RecA2 domain of nsp13 (Fig. S2). This proved to be a consistent finding, as we followed up with docking of the nsp9 dimer to several available structures of nsp13, capturing a variety of conformations of this protein (J. Chen, unpublished results). Notably, the position of nsp9 on nsp13 is such that it is ideally situated to interact with the 5′ end of the ssRNA as it exits the helicase RNA-binding groove.

**Figure 2:**
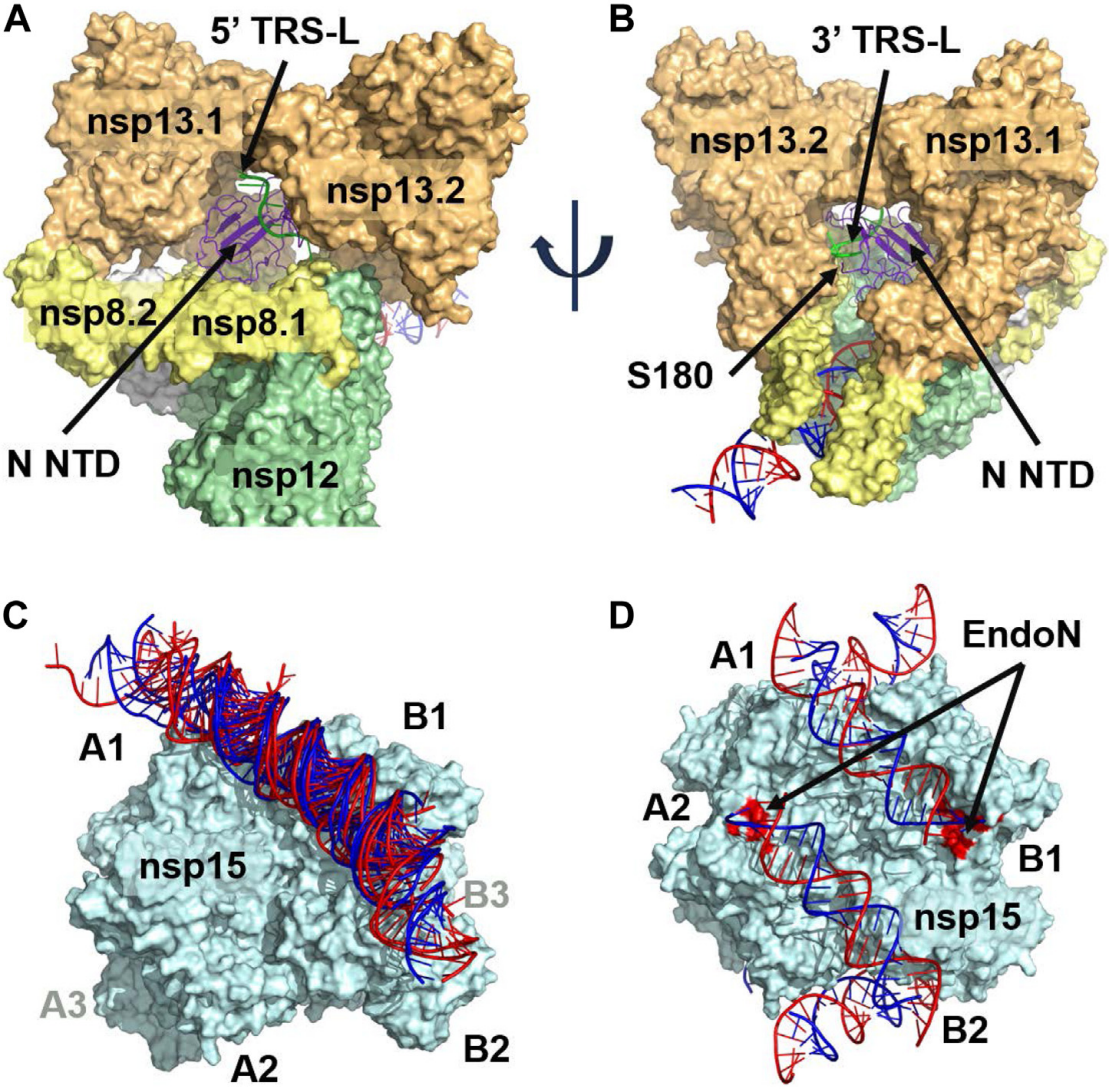
**Significant findings from protein–protein docking.***A* and *B*, the N NTD bound to a TRS 10 nt oligo (PDB: 7ACT) docks into the void between the two nsp13 subunits of the polymerase complex. The TRS oligo is positioned over the polymerase active site, parallel to the template, with its 5′ end exposed on the entrance side of the polymerase and its 3′ end exposed on the exit side. The C-term S180 residue of the N NTD is also exposed on the exit side of the polymerase, indicating full-length N could bind to the complex unobstructed. *C*, multiple examples of dsRNA docked across the nsp15 hexamer, spanning subunit A1 to B1/B2. *D*, six dsRNA double helices can be symmetrically arranged around the hexamer: three directed from A → B, and three directed in an antiparallel fashion from B → A. Each dsRNA passes over an EndoN active site, colored in *red*.

Additionally, we found that the NTD of the N protein, as bound to a 10-nt oligo corresponding to the TRS-L of the SARS-CoV-2 genome, binds robustly between the two nsp13 subunits of the polymerase complex (Fig. 2, *A* and *B*), as all 30 of the returned binding modes were variations on this same interaction. This positions the TRS-L above the Pol active site, having implications for template switching during transcription. To emphasize this point, the top scoring docking pose orients the TRS-L RNA parallel to the polymerizing template strand, with the 5′ end on the entrance side of the Pol active site and the 3′ end on the exit side. The C-terminus (C-term) of this domain, as defined by residue S180, is also well exposed to solvent on the exit side of the polymerase, implying that full-length N protein could bind unimpeded. Interestingly, we found that structures of the N protein that are either apo or dsRNA binding (PDB:7ACS) (31) do not dock to this site. Only the structure with the cocrystalized TRS-L segment binds here, which is likely due to its more spherical shape.

### Nsp15-dsRNA interaction

Having completed this initial survey of potential binary protein–protein interactions, we considered the possibility that nsp15 retained its hexameric form in the RTC. Docking of the nsp15 hexamer to the polymerase complex did not identify any direct interaction between the proteins, but instead revealed a specific interaction between nsp15 and dsRNA that was common to all of the top poses. We followed up with docking of isolated dsRNA to the nsp15 hexamer and again found this particular binding mode was dominant (Fig. 2*C*). The RNA runs from one face of the nsp15 hexamer to the other and is in contact with three subunits (*e.g.*, A1, B1 and B2). As shown in Fig. S3, it has direct interactions with at least eight basic residues (K110, R135, and K149 from subunit A1, K316, K319, K334, and K344 from B1, and K12 from B2) and passes over one of the EndoN active sites (on subunit B1). An appealing aspect of this finding is that we can arrange six dsRNAs around the hexamer without interference (Fig. 2*D*): three run from face A to face B and three run in the antiparallel direction from face B to face A. Each passes over a different EndoN site, suggesting to us that nsp15 could act as the core of the RTC, facilitating multiple replication cycles and directing RNA from the polymerase of one subunit to distal subunits that perform capping.

### Nsp15-nsp14/nsp10 interaction

With our determination that nsp15 interacted with dsRNA but did not appear to interact directly with nsp12, we considered the possibility that another protein, which interacted with dsRNA, could sit between the two. An initial hypothesis we had with respect to proofreading was that the nsp14 ExoN interacted with the dsRNA as it exited the polymerase. We arrived at this conclusion based on an analysis of ExoN locations relative to the polymerase active site in various DNA polymerases. Notably, bacterial DNA polymerase I (*e.g.*, the *E. coli* Klenow fragment) (42) and mammalian DNA polymerase γ (43) have the ExoN site located below the exiting dsDNA. Other potential locations of the ExoN domain, such as that seen in mammalian DNA polymerase δ (44), are precluded by the known positions of nsp7, nsp8, and nsp13. Bolstering this hypothesis, the coronavirus ExoN has been shown to have a preference for dsRNA or hairpin ssRNA substrates over unstructured ssRNA (11). Furthermore, a cocrystal structure of the similar Lassa ExoN allowed us to construct a model of how the nsp14 ExoN interacts with dsRNA (45). This model has since been confirmed by the recently published cryo-EM structure of nsp14/nsp10 interacting with a hairpin RNA, which was not available at the time this work was done (46).

With this in mind, we considered that nsp14 could interact with the dsRNA as it is bound to the nsp15 hexamer. However, docking of nsp14 to this complex did not produce any meaningful poses. Manual exploration suggested a potential site of interaction where the ExoN was located under the dsRNA on the face opposite to the EndoN site (following the above example, with the EndoN site on B1, the ExoN could be positioned to interact with the RNA on A1). Intriguingly, this location allowed us to simultaneously position the zinc fingers of nsp10 over an antiparallel dsRNA just after it passes over another EndoN site (on A2). As attractive as this location was, the issue with this binding pose was that the MTase domain was not ideally situated with respect to nsp15. A similar situation arose when attempting to find an interaction between the ExoN and the exiting dsRNA of the polymerase complex. A location that functionally made sense for the ExoN was not ideally positioned with respect to the MTase domain.

As described above, a sizable limitation of the protein–protein docking method employed is that it assumes the proteins have minimal flexibility and does not sample alternative conformations. The available structures of SARS-CoV nsp14 suggest at least some limited flexibility between the two domains exists (6, 16). As they are linked by a 14-residue loop (residues 286–299), we considered the possibility that there may be greater flexibility between these two domains, an hypothesis supported by a recently published molecular dynamics (MD) study of SARS-CoV-2 nsp14 (17) and made more likely in the context of protein–protein perturbations. Thus, given our assessment that the ExoN could interact with the dsRNA on both the polymerase complex and the nsp15 hexamer, and that it could potentially adopt a range of conformations with respect to the MTase, we decided to handle the two domains separately, first optimizing the position of the ExoN (residues 1–285) and nsp10 on nsp15 and then docking the MTase (residues 300–526).

**Figure 3:**
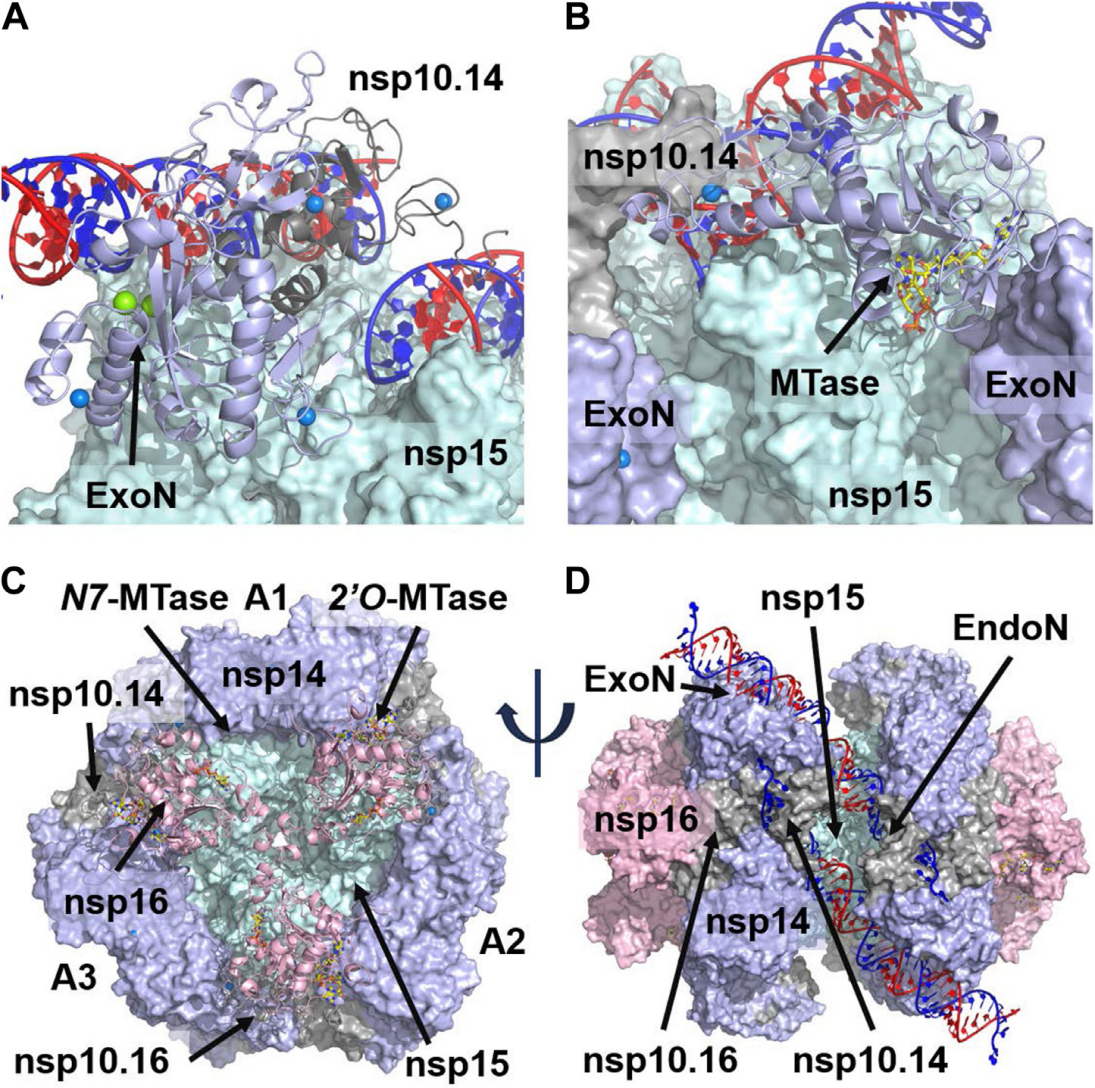
**Formation of the nsp15/nsp14/nsp16/nsp10 complex.***A*, the nsp14 ExoN NTD and nsp10 were manually positioned to interact with the dsRNA on the nsp15 hexamer, following the observed Lassa ExoN interaction with dsRNA. Nsp10 is positioned such that its two zinc fingers are over an antiparallel dsRNA, just past the nsp15 EndoN site. Six ExoN/nsp10 subunits can be arranged around the nsp15 hexamer. *B*, the nsp14 MTase CTD was docked to the nsp15/ExoN/nsp10 hexameric complex. The CTD zinc finger is positioned over an antiparallel dsRNA, opposite the nsp10 associated with another nsp14 subunit. The binding mode reflects a significant conformational change between the two domains of nsp14. *C*, Nsp16/nsp10 is docked to the nsp15/nsp14/nsp10 hexameric complex. Nsp10 is positioned between two nsp14 subunits, while three nsp16 subunits meet in the middle of the nsp15 trigonal face. *D*, the full nsp15/nsp14/nsp16/(nsp10)_2_ hexamer.

We optimized the structure of ExoN/nsp10 interacting with nsp15 and dsRNA as described above (Fig. 3*A*) and arranged six subunits symmetrically around the hexamer. We then docked the MTase domain to this complex and observed a compelling binding mode in which the MTase rotated approximately 180° with respect to its X-ray structure conformation (Fig. 3*B*). The interface with nsp15 had excellent shape complementarity and established as many as five salt bridges, six hydrogen bonds, and a cation–pi interaction. The C-term zinc finger interacted with another antiparallel dsRNA as it passed over an EndoN site (on A1), while the C-term helix (residues 515–526) sat in the major groove of this same dsRNA. This set up a situation where three zinc fingers (two from nsp10 associated with another subunit and one from the nsp14 CTD) converged around the dsRNA just past the EndoN site. From this starting point, the nsp14/nsp15 structure was completed by building back in the 14-residue loop connecting the two nsp14 domains. Once fully optimized, the six nsp14/nsp10 subunits formed trimeric rings around each face of the nsp15 hexamer. A summary of the residues defining the nsp15/nsp14 interface is presented in Table S1.

### Nsp16/nsp10-nsp14-nsp15 interaction

After optimizing the nsp15 and nsp14/nsp10.14 interaction, we used protein–protein docking to investigate possible binding locations for nsp16/nsp10.16. From this, we identified an unambiguous site in which nsp10.16 was positioned between the nsp14 ExoN domain of A1 and the MTase domain of A2 (Fig. 3*C*). Three such binding sites exist on each face of the hexamer, leading to the three nsp16 subunits lightly contacting in the center of each face. Key interactions included as many as six salt bridges and coordination of nsp16 D102 to the nsp14 ExoN zinc finger at C206, C209, and C225. As docking was performed without the nsp10.16 N-term residues 1 to 23 and C-term residues 132 to 139, these were subsequently built in during optimization. The flexible nsp10.16 N-term helix (residues 11–20) easily fit into a pocket formed by the nsp14 ExoN and its associated nsp10.14 subunit. With the placement of both nsp14/nsp10.14 and nsp16/nsp10.16, a ring of three nsp14/nsp16/(nsp10)_2_ subunits cap one face of the nsp15 hexamer, with an equivalent ring on the opposite face. Each of these rings establishes an interaction between its three ExoN domains and three dsRNA helices, while also positioning clusters of zinc fingers around the three antiparallel dsRNA helices just above the EndoN sites (Fig. 3*D*).

The significance of this placement of nsp16/nsp10.16 with respect to nsp15/nsp14/nsp10.14 is that it puts both MTase sites near each other, an outcome consistent with their roles in capping. It also adds an additional pair of zinc fingers to interact with the dsRNA just past the EndoN site. In total there are six of these in close proximity (two from nsp10.14, two from nsp10.16, one from nsp14 ExoN, and one from nsp14 MTase). Interestingly, a narrow channel lined with basic residues is carved out by this arrangement of the proteins that appears suitable to accommodate ssRNA (basic residues include R80, H81, R83, R179, R204, R212, and R277 from nsp14; K25, K28, K113, and K124 from nsp10.14; and H242 from nsp15). It runs from the EndoN site to the trigonal face accommodating both MTase sites. Given the dsRNA steric blockade also created by these proteins, we concluded this was the site of strand separation. Without the need for a helicase, the model suggests that strand separation is facilitated by the zinc fingers, three of which (two from nsp10.14 and one from nsp14 MTase) act to direct the 3′ strand away from the complex, while two others (one from nsp10.16 and one from nsp14 ExoN) direct the 5′ strand into the basic channel. From there, the 5′ strand is funneled to the mRNA capping sites (nsp14 MTase and nsp16 MTase). We built a model of RNA following these two paths to illustrate the point.

### Nsp12/(nsp8)_2_-nsp14/nsp15 interaction

As discussed above, we propose the most likely site for the ExoN and the polymerase to interact is on the exit face of nsp12, with the ExoN sitting below the dsRNA. This position of the ExoN relative to the polymerase active site has precedent in the *E. coli* DNA polymerase I Klenow fragment (42), although the proposed binding orientation is not strictly identical. But as with nsp14/nsp15, docking of existing structures of nsp14 was unsuccessful in finding a suitable binding mode. The ExoN domain could be manually positioned to a satisfactory degree, but the MTase domain could not be properly placed.

**Figure 4:**
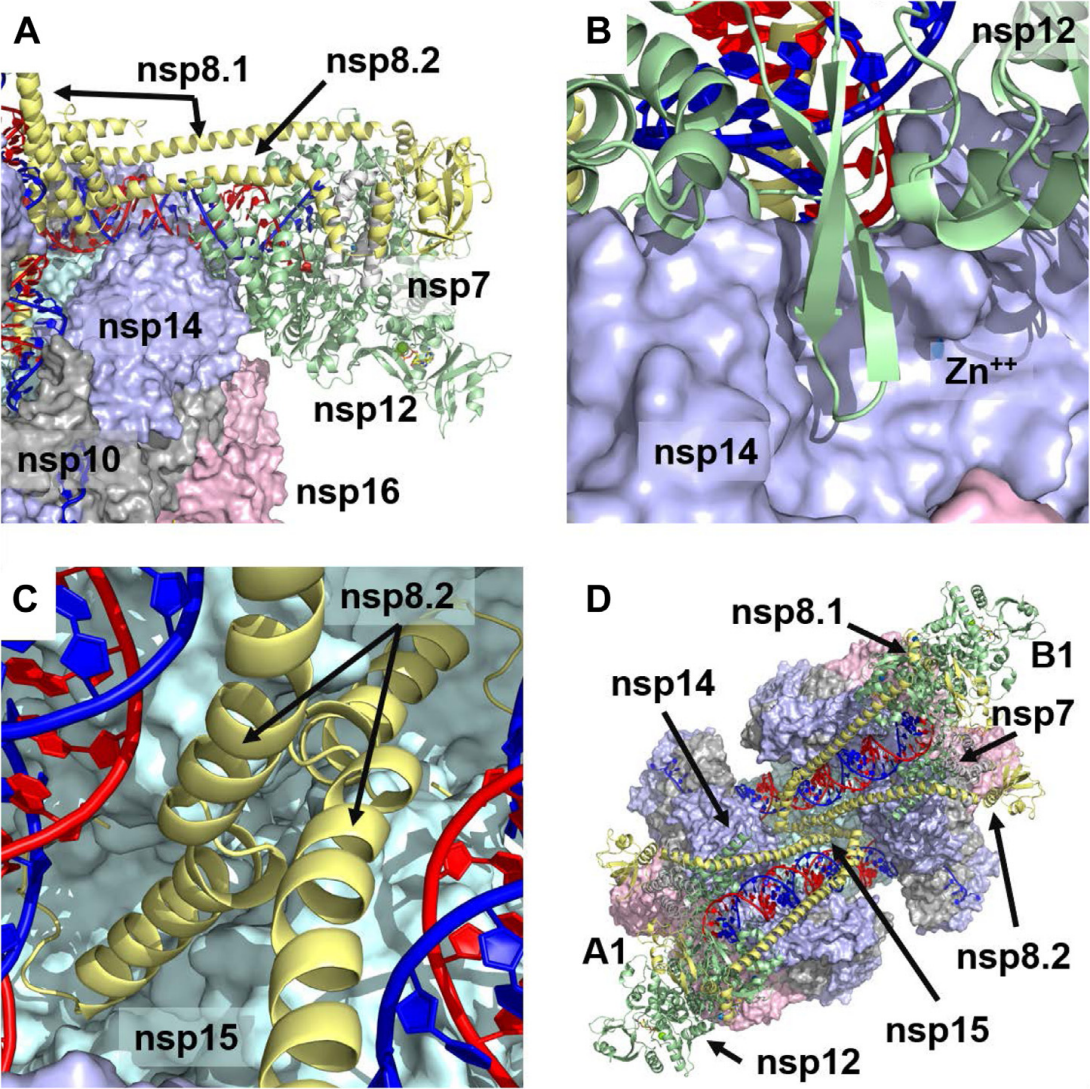
**Binding of the polymerase to the nsp15/nsp14/nsp16 complex.***A*, binding of the polymerase is largely through nsp12 interactions with the conformationally altered face of nsp14. Much of this binding comes from the C-term helices of nsp12 (residues 855–923) interacting with the MTase domain of nsp14. *B*, the nsp12 beta hairpin (residues 815–831) sits in the cleft between the two domains of nsp14, in close proximity to a zinc finger. *C*, the short N-term helix of nsp8.2 (residues 12–28) extends to interact with nsp15, with the N-term residues (1–11) sitting under the dsRNA just ahead of the EndoN site. *D*, view of a pair of polymerases bound to the nsp15/nsp14/nsp16 complex. One polymerase complex is associated with the A1 nsp14 subunit, while the other is associated with the B1 subunit. Their nsp8.2 subunits meet in the middle where they interact with nsp15.

This situation changed dramatically with the conformational change to nsp14, facilitated by binding to nsp15. This new conformation makes a significantly different surface available for binding to nsp12. With additional steric constraints coming from the nsp8 helices of the polymerase complex, which extend along the exiting dsRNA, a single binding mode between nsp12 and nsp14/nsp15/nsp16 became the only choice and was further optimized. This binding position fuses the dsRNA of the nsp14/nsp15/nsp16 complex with the dsRNA of the polymerase complex, placing the ExoN directly below the RNA as it exits nsp12 (Fig. 4*A*). The MTase domain binds to the surface of nsp12 created by the helical C-term residues from 855 to 923, establishing multiple hydrogen bonds and salt bridge connections. The unusual beta hairpin (815–831) that protrudes from nsp12 below the dsRNA is positioned in the cleft between the two nsp14 domains (Fig. 4*B*). Notably, D825 on this beta hairpin is in proximity to another nsp14 zinc finger, which sits below the ExoN active site. This complex was optimized, allowing additional flexibility for the poorly resolved nsp12 C-term residues 917 to 929. A summary of the residues comprising the nsp12/nsp14 interface is presented in Table S2.

When arranging additional polymerase complexes around the hexamer, we then focused on refining the position of the nsp8 N-term helices, which extend over the dsRNA. These helices are composed of a short helix (residues 12–28) and a long helix (residues 32–97), which are seen to fold back into a bundle *via* a short connecting loop (residues 29–31). The helices of nsp8.1 did not appear to provide any new contacts with other proteins of the complex, stopping just short of touching nsp14 on the opposite face. However, nsp8.2 appeared to have a close contact with nsp8.2 coming from the opposite face (*e.g.*, subunits A1 and B1). This interaction occurs in the center of the complex at the loop connecting the long and short nsp8 helices (residues 29–31). We initially considered optimizing the structure in this state; however, given direct binding of MERS nsp8 to nsp15 has been observed (27), as has cellular colocalization of SARS nsp8 and nsp15 (37), we considered the potential for a greater interaction between nsp8.2 and nsp15, with the most likely point of contact coming from the short helix and N-term residues. We thus docked the short nsp8 N-term helix to the nsp15 hexamer and identified a site in the center of the complex, which would be consistent with a nearly linear extension of the short nsp8 helix from the longer nsp8 helix. Optimization of this extended state positioned the N-term residues on nsp8.2 under the dsRNA just prior to it passing over the EndoN site (Fig. 4*C*). This set up a situation where the two nsp8.2 helices coming from opposite faces crossed in the center of the complex. Optimization of this crossing was facilitated by symmetric interactions between nsp15 R138 and nsp8 D30 and E32 on each subunit. This structure established a framework of pairs of polymerase complexes binding through nsp14 to the hexameric nsp15 complex (Fig. 4*D*). Three such pairs can be arranged around the hexamer.

### Arrangement of nsp13

**Figure 5:**
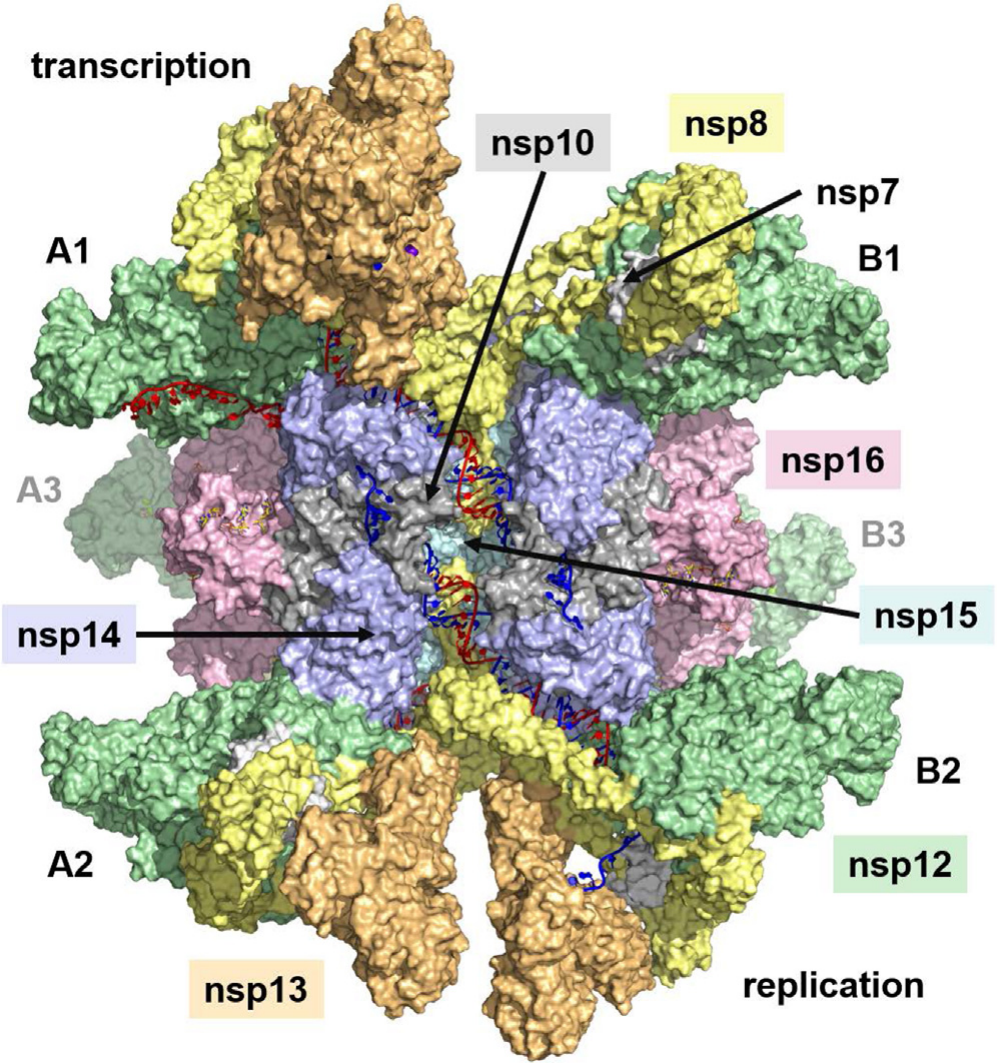
**The complete replication/transcription complex, with a stoichiometry of six nsp15, six nsp14, six nsp16, six nsp12, six nsp13, six nsp7, 12 nsp8, 12 nsp10, and 2 N proteins.** The six nsp13 subunits are arranged across nsp12 pairs in 2/0 (A1/B1), 1/1 (A2/B2), and 0/2 (A3/B3) stoichiometries. The polymerase complexes with two associated nsp13 subunits (A1 and B3) bind the N protein with the TRS-L oligo and are responsible for template switching during negative-strand synthesis (transcription). The two polymerase complexes with a single nsp13 subunit (A2 and B2) are responsible for replication.

The position of nsp13 in the complex is already established by multiple cryo-EM structures, which show that two nsp13 subunits can coordinate to each nsp12 polymerase through the nsp8 subunits (5, 32, 36, 47). However, it is unclear if that will occur in the larger complex. Polymerases with a single nsp13 subunit associated, as well as structures with no associated nsp13, have been observed (32). Considering that nsp12, nsp13, nsp14, nsp15, and nsp16 stem from a single polyprotein and are expected to be generated in equal quantities, it is reasonable to propose that six nsp13 subunits associate with the hexameric RTC. This is reinforced by the pairwise arrangement of nsp12 subunits on the complex. The proximity of these subunits, in and of itself, does not lead to clashes, such that four nsp13 subunits could theoretically be accommodated on a single pair of polymerases (12 in total on the complex). However, as discussed below, we expect that the N-protein bound nsp13 dimer will coordinate the bulky 5′-UTR RNA, which appears unlikely for a complex saturated with nsp13 subunits. If the number of nsp13 subunits is limited to two for each polymerase pair, no such spatial limitations exist. Thus, the two nsp13 subunits can be arranged across polymerase pairs either by situating both on a single polymerase or distributing one on each. We propose that six nsp13 subunits are distributed among the three polymerase pairs as follows: two on A1 and none on B1; one on A2 and one on B2; and none on A3 and two on B3 (Fig. 5 and Video S1). The two polymerases that coordinate two nsp13 subunits each (A1 and B3) can bind the N protein, positioning the TRS-L above the Pol active site. These two nsp12 subunits would thus be in a configuration appropriate for transcription. The two polymerases that coordinate only a single nsp13 subunit (A2 and B2) would not bind the N protein and only be capable of replication. Thus, this arrangement would support four simultaneous polymerization events: two driving negative-strand synthesis with template switching (transcription) and two driving positive-strand replication.

Our optimized model, which is 343,104 atoms (including protons), is available in PDB form in the Supplementary Material. We should note that given our finding that nsp9 associates with nsp13, it would be appropriate to include nsp9 in this model as well. We have opted not to include it at this time, in that further work is needed to understand its role, particularly as to how it relates to the dynamics of nsp13.

## Discussion

### Overall organization

Multiple studies have sought to map the interactions between the viral proteins that comprise coronavirus replication and transcription complexes (27, 37, 38, 39, 40). Details have varied, but the overall picture that has emerged is that the complex is formed from the proteins of *ORF1ab* and the nucleocapsid. Included among these proteins is nsp15, which is often viewed as nonessential, because studies of murine hepatitis virus (MHV) showed that inactivating mutations within the EndoN active site are not lethal (48). However, these same studies demonstrated that a mutation outside of the EndoN active site, thought to cause misfolding of the protein, is in fact lethal to the virus. This suggests that nsp15 could play a structural role beyond its enzymatic activity. Furthermore, three studies in particular have highlighted the associations of nsp15 with other proteins of the RTC. Athmer *et al.* (37) studied *in situ* tagged MHV nsp15, finding that it strongly colocalized with nsp8 and nsp12 during active infection. Zhang *et al*. (27) used microscale thermophoresis (MST) to confirm the direct binding of MERS nsp8, but not nsp12, to nsp15. Finally, a recent study by Xu *et al*. (40) used compartmentalization of protein–protein interactions in cells (CoPIC) to identify 47 binary interactions between the proteins that make up the SARS-CoV-2 RTC. The authors specifically highlighted the interactions of nsp15, which included nsp10, nsp14, and nsp16.

With the nsp15 hexamer forming the central core of the RTC in our model, we found that three subunits each of nsp14/nsp10 and nsp16/nsp10 combine to form trigonal caps, two of which bind to opposite faces of the nsp15 hexamer. This leads to an overall nsp15:nsp14:nsp16:nsp10 stoichiometry of 6:6:6:12. To achieve this, nsp14 undergoes a significant conformational change where the MTase domain rotates approximately 180° relative to its X-ray structure conformation. While a small degree of conformational flexibility of this protein has been observed in the available SARS-CoV X-ray crystal structures, a much larger conformational change has only recently been demonstrated *via* MD simulations (17). The conformational change we observe here is energetically unfavorable for isolated nsp14, but is compensated by binding to nsp15 and nsp16.

The conformational change that nsp14 undergoes upon complexing with nsp15 and nsp16 enables its binding to nsp12. Much of the nsp12-nsp14 interaction occurs on the newly exposed face of the nsp14 MTase domain and within the cleft between the MTase and ExoN domains. The ExoN domain is positioned to interact with dsRNA as it exits the polymerase, providing the foundation for a model of proofreading as described below. The arrangement of the polymerase subunits around the complex can be viewed as three sets of pairs (labeled A1/B1, A2/B2, and A3/B3). Within each pair, the nsp8.1 N-term helices are seen to cross in the center of the complex, extending to gain an additional interaction with nsp15.

Existing cryo-EM structures of the polymerase complex would suggest that up to 12 nsp13 subunits could saturate the RTC. However, we suggest that the actual number of nsp13 subunits is likely six, retaining the expected molar ratios from the *ORF1ab* polyprotein. These can be arranged either with two nsp13 subunits on one nsp12 subunit and none on the paired nsp12 subunit, or with one each on both paired nsp12 subunits. As we showed that the N protein NTD binds to the polymerase complex between two nsp13 subunits, positioning the TRS-L RNA above the polymerase active site, this situation would be suitable for negative-strand synthesis, which involves template switching at the TRS. With no such requirement for positive-strand synthesis, the polymerase complexes with a single nsp13 subunit may be suitable for full-genome replication. While the distribution of the nsp13 subunits could prove to be dynamic, we propose an ideal arrangement of the nsp13 subunits across the RTC in which two nsp12 subunits coordinate a pair of nsp13 subunits each, two nsp12 subunits coordinate a single nsp13 subunit each, and the other two nsp12 subunits coordinate no nsp13. Such an arrangement would accommodate four simultaneous polymerization events, two suitable for positive-strand synthesis and two for negative-strand synthesis.

**Figure 6:**
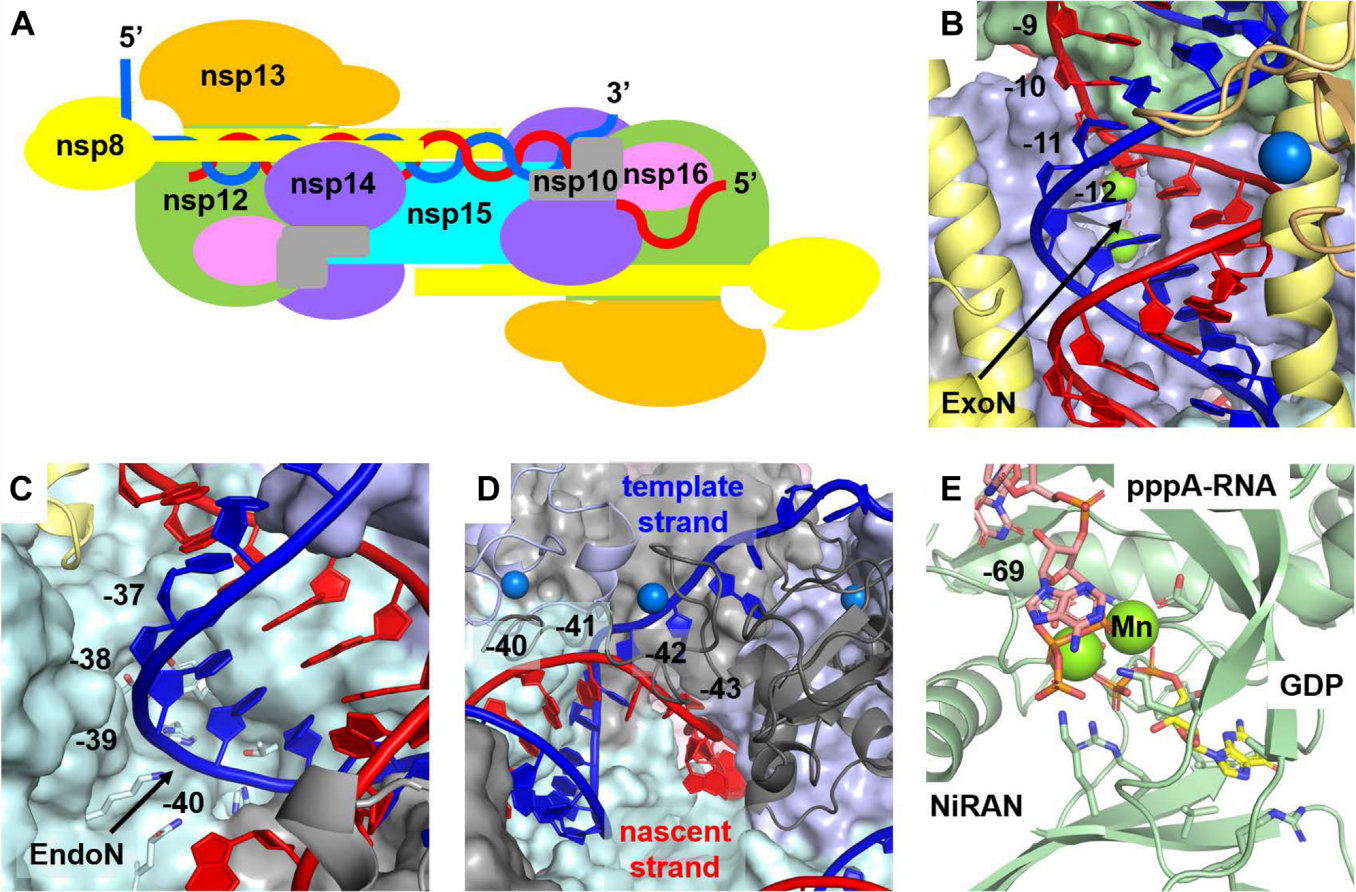
**Details of some key functions.***A*, schematic representation of the RNA path. dsRNA makes its way from the nsp12 polymerase, across the nsp14 ExoN and nsp15 EndoN. It is separated into template (*blue*) and nascent (*red*) strands at nsp10, and the nascent strand is directed to the NiRAN and two MTase sites. *B*, detail of dsRNA exiting the polymerase and passing over the ExoN. Nucleotides of the nascent strand are numbered starting from the 3′ primer position (−1), where nucleotide −12 is seen to pass over the ExoN site. The dsRNA is expected to shift into the ExoN active site when encountering a prematurely terminated nascent strand. *C*, detail of the dsRNA passing over the EndoN site, where nucleotides −39 and −40 of the template strand are best situated for potential cleavage. *D*, detail of strand separation occurring at the convergence of two zinc fingers from nsp10.14 and one from nsp14 CTD. Strand separation occurs across the base pairs −41 to −43. The template strand is directed away from the complex, while the nascent strand is funneled down to the capping sites. *E*, detail of the NiRAN site. The first capping step occurs when the NiRAN site transfers GDP to the 5′ pppA-RNA, releasing a pyrophosphate. This occurs at the terminal nucleotide −69 in our model.

The resulting structure, constructed and optimized largely through first principles, provides a detailed atomic level model of coronavirus RNA replication and transcription. It suggests an efficient process in handling the viral RNA. As depicted schematically in Figure 6*A*, upon exiting the Pol active site, dsRNA passes over the ExoN site of nsp14 and then the EndoN site of nsp15. At this point, it encounters a collection of proteins, which includes nsp14, nsp10.14, and nsp10.16. The close proximity of several zinc fingers leads to strand separation, where the template strand is directed away from the complex, while the nascent strand is funneled to sites responsible for mRNA capping. We describe below the implications of the structure on the major known functions of the RTC, including proofreading, mRNA capping, and template switching.

### Proofreading

Immediately upon exit from the polymerase, the newly formed dsRNA encounters the nsp14 ExoN (Fig. 6*B*). This site is one complete helical turn from the polymerase active site. In an actively elongating state, the dsRNA is not expected to engage with the ExoN catalytic Mg^++^ ions, as it is prevented from doing so by steric interactions between the nascent strand and nsp14 P141 and G250 on loops that flank the active site. Examination of the homologous Lassa ExoN structure with bound dsRNA (45) indicates that dsRNA can engage the ExoN site only at the 3′ terminal nucleotide. The recent cryo-EM structure of a hairpin RNA bound to nsp14/nsp10 (published after completion of this work) (46) confirms this picture and further emphasizes selectivity for cleavage of mismatched bases. The 3′ terminus removes the steric constraints coming from the nascent strand, while the mismatch allows the RNA to collapse into the active site. This suggests a scenario for proofreading in which a pause in nucleotide incorporation following a mismatched incorporation event would be subject to translocation of the prematurely terminated RNA to the ExoN site. The 3′ terminated RNA, now free of steric constraints, shifts into the active site to cleave the 3′ mismatched base. While we are not aware of a precedent for RNA translocation in the absence of incorporation, recent molecular tweezers studies of the dynamics of nsp12/nsp7/nsp8 nucleotide incorporation indicate that translocation is facilitated by thermal activation rather than by a power stroke (49). It is proposed that forward translocation is then halted by NTP binding and closure of the active site. It is unclear how the other proteins in the complex influence translocation or how misincorporation alters this picture.

Once the 3′ terminal nucleotide is removed, the RNA would need to be reset to continue polymerization. Cryo-EM structures have shown that the 5′ template overhang sits in the RNA binding groove of the nsp13.1 helicase (36, 47). But nsp13 is an SF1B helicase, with a polarity (5′ → 3′) that runs opposite to that of the polymerase (3′ → 5′), setting up a tug of war between the two. A solution to this conundrum has been suggested by a recent series of structures (J. Chen, unpublished results) which reveals nsp13 can adopt what appears to be an inactive state. In this state, the 1B domain undergoes a large conformational shift, opening up the RNA-binding groove. This state is similar to the inactive state observed in the SF1B Pif1 DNA helicase from *Bacteroides* spp, where a rotation of the 2B domain was demonstrated to regulate activity of the helicase (50). Thus, in its inactive form, polymerization can proceed unimpeded. However, when the protein is activated, the template would be expected to reverse course, leading to backtracking. While this concept of backtracking was previously suggested to initiate proofreading (47), here we suggest that it is used to return the RNA from the ExoN to the Pol active site.

How backtracking would be triggered is unknown. Dynamics simulations have demonstrated interconversion between the states of nsp13.1, with the less stable inactive state being trapped by nsp13.2 (J. Chen, unpublished results). However, other proteins such as nsp9, which was shown here to bind to the RecA2 domain of nsp13, could also serve to regulate these states. With respect to proofreading, we suggest that a shift in the position of the RNA as it engages the ExoN is sensed by the coordinating nsp8 helices. These helices have proven themselves to be highly flexible and may serve to transmit changes in the RNA position to the nsp13 zinc binding domains (ZBDs). When the RNA is out of position, nsp13 is activated, causing the RNA to reverse course. Once the RNA is returned to the polymerase active site, nsp13 resumes its resting inactive state, and RNA synthesis can continue.

### Endonuclease activity

The nsp15 endonuclease is less well characterized than some of the other viral enzymes. It shows a preference for cleavage at uridine (51), and dsRNA is a better substrate than ssRNA. Recent work by Baker *et al.* (9, 52, 53) suggests the EndoN acts on the 5′ poly-U tail of the negative strand to avoid host immune responses. Indeed, while positive-strand 3′ poly-A tails can reach lengths of 100 to 130 nt’s (54), negative-strand 5′ poly-U tails are significantly shorter (9–26 nt’s). As with the nsp14 ExoN site, the dsRNA, which crosses nsp15 from one face to the other, appears to be sterically constrained from engaging the EndoN site (Fig. 6*C*). The template RNA is positioned directly above the EndoN site and appears more likely to be the substrate than the nascent strand, but it is held aloft by basic residues across the length of the complex. A scenario in which the EndoN site might be engaged would be once synthesis is complete and the blunt end of the dsRNA has traveled far enough across the nsp15 hexamer that it would no longer be supported by the full complement of basic residues. A shifting of the remainder of the dsRNA would occur, allowing it to engage the EndoN active site. The model suggests this would be ∼10 nts from the 5′ end of the template. This would be consistent with observations that nsp15 acts on the 5′ poly-U tail of the negative-strand during positive-strand synthesis. Thus, once positive-strand synthesis is complete, the negative-strand poly U tail would be truncated.

### Strand separation

The proteins of the RTC feature multiple zinc fingers, whose roles remain unclear. In total, there are 12 zinc fingers for each unit of the hexamer: three on nsp13, three on nsp14, two on nsp12, and two on each of the nsp10 subunits. Of these, two may be important for protein–protein binding: the nsp14 Zn^++^ at C206/C209/C225/H228 coordinates to nsp16 D102, and the nsp14 Zn^++^ at H256/C260/H263/C278 coordinates to nsp12 D825. But within the complex, we find a particularly interesting set of six of these zinc fingers, coming from four different subunits that are positioned in close proximity to the EndoN active site. Two of these come from nsp10.14 and two from nsp10.16, while another two come from two distinct subunits of nsp14: the Zn^++^ at C206/C209/C225/H228 in the ExoN domain and the Zn^++^ at H486/C451/C476/C483 in the MTase domain.

Following the trajectory of the dsRNA as it crosses the nsp15 hexamer, nsp14 and nsp10.16 form a barrier, which prevents the dsRNA from advancing beyond the EndoN site. But this area, rich in both basic residues and the zinc fingers detailed above, creates well defined pathways to separate the template and nascent strands (Fig. 6*D*). The nsp14 CTD zinc finger and one of the nsp14.10 zinc fingers sit above the nascent strand, distorting its path. The template strand is sterically prevented from continuing in its dsRNA trajectory by two N-term helices of nsp10.16 and is directed between the two nsp10.14 zinc fingers away from the complex. The nascent strand is then directed into a channel lined with basic residues primarily coming from nsp14 ExoN. It encounters two additional zinc fingers (the nsp14 ExoN zinc finger and one of the zinc fingers from nsp10.16). This channel funnels the nascent strand into the region where the capping sites (the nsp12 NiRAN site and the two MTases) are found. This model suggests that the nsp13 helicase is not involved in strand separation. It is more consistent with strand separation observed in negative sense viruses such as influenza (55).

### mRNA capping

The capping of the 5′ end of the positive sense RNA occurs through a series of steps that involve transfer of a G, followed by two methylation events (pppN-RNA → GpppN-RNA → m^7^GpppN-RNA → m^7^GpppNm-RNA) (56, 57). Methylation is facilitated by the nsp14 and nsp16 MTases, but the first step is less clear. There is some evidence that both nsp13 and the NiRAN site on nsp12 are involved (3, 5, 8). Following conventional mRNA capping mechanisms, the general assumption is that nsp13 acts as a 5′ RNA triphosphatase (NTPase), converting pppN-RNA to ppN-RNA, and the NiRAN site acts a guanylyltransferase (GTase), transferring GTP to ppN-RNA and releasing pyrophosphate. However, while pppN-RNA has been shown to be dephosphorylated by the nsp13 NTPase site, it is a mediocre substrate, being completely inhibited by cellular level concentrations of ATP (8). In our model, Nsp13 is also not in the general vicinity of the other enzymatic sites linked to capping, making it unlikely to interact directly with the RNA during capping. On the other hand, GTP is a good substrate for nsp13, suggesting there should be relatively high local concentrations of GDP (8). Thus, we suggest that pppN-RNA is not dephosphorylated prior to the first capping step, but instead the NiRAN site facilitates the transfer of GDP, releasing pyrophosphate, a mechanism more consistent with rhabdoviruses (57).

Interestingly, the NiRAN site appears to have two functions. It is capable of nucleotidylating proteins with only a moderate preference for UMP (4) and has also been shown to act on RNA (5). Should it be responsible for the first step in capping, this would necessarily be G specific. While several cryo-EM structures of nsp12 have shown binding of nucleotides at the NiRAN site in a non-base specific manner, a recent cryo-EM structure with the guanosine analogue inhibitor, AT-527, identified a novel binding mode of the diphosphate form of the inhibitor in the NiRAN site that appears to specifically recognize its base (58). With this as a starting point, we modeled the approach of pppA-RNA to the GDP-occupied NiRAN site (Fig. 6*E*).

After strand separation, the nascent strand is fed into the nsp15 trigonal face, where all three proposed capping sites are found (Fig. S4). Charting its most likely initial pathway *via* conformational sampling, the RNA passes by three additional zinc fingers (a second nsp14 ExoN zinc finger and two on nsp12) before dropping down into the NiRAN active site. Notably, there is currently some question as to whether the two nsp12 metal-binding sites indeed coordinate Zn^++^ or alternatively coordinate Fe-S clusters (59). The current model is agnostic with respect to this question, emphasizing only that there appears to be some interaction between the nascent strand ssRNA and these metal sites that helps direct the RNA to the NiRAN site. There, the pppN-RNA triphosphate binds to the same pair of catalytic Mn^++^ ions that bind to GDP. The orientation is such that a linkage between the β-PO_3_ of GDP and the α-PO_3_ of pppN-RNA would be formed upon release of pyrophosphate. It is unclear if this occurs in a single step or if a nucleotidylated protein intermediate is involved.

From this point, the GpppN-RNA would continue to the nsp14 *N7*-MTase to become m^7^GpppN-RNA and then on to the nsp16 *2′-O*-MTase to become m^7^GpppNm-RNA (Fig. S5). Further simulations are required to identify the probable path the RNA would take. There are several possible trajectories, as more than one site for each of these MTases is accessible, and there are multiple areas across the entire capping region that are rich in basic residues to guide the RNA.

### Transcription

Discontinuous transcription is an unusual process in which a large segment of the template RNA is skipped over to create a set of nested subgenomic mRNAs with a common 3′ end of varying lengths, all terminated with the same 5′ leader (41). The shift in template is triggered when the TRS element is reached during negative-strand synthesis. As shown in Figure 1, this sequence of 7 to 10 nts is found several times throughout the genome: once in the 5′-UTR (referred to as TRS-L) and then preceding the starts of known ORFs (referred to as TRS-B). When polymerization reaches the TRS-B, the newly synthesized complementary negative strand may recouple to the same template sequence in the TRS-L, completing synthesis of the 5′ leader and skipping over the regions in between. The N protein has been thought to be involved in this process of recoupling, in that its NTD has been shown to specifically bind to the TRS sequence and is essential to transcription (13, 60). Yet a detailed picture of the mechanics of template switching is largely a mystery.

Here we showed *via* protein–protein docking that the N protein, when bound to the TRS oligo, positions itself between two nsp13 subunits over the polymerase active site. The orientation is such that once the complementary sequence is synthesized, the nascent strand could potentially recouple to this parallel template. We envision several factors that would allow this template switch to happen.

**Figure 7:**
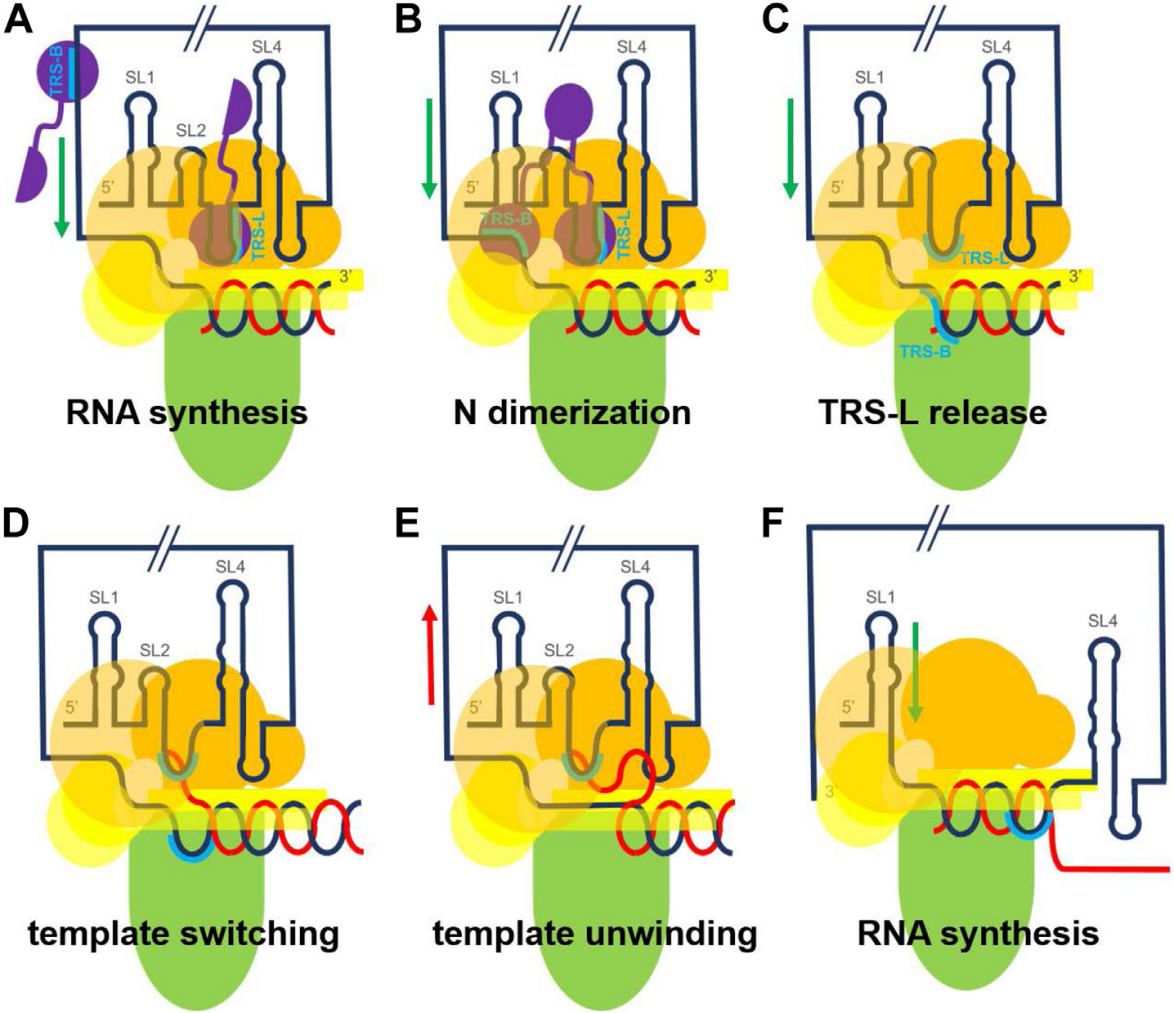
**Model of template switching [nsp12 (*green*), nsp13 (*orange*), nsp8 (*yellow*), N protein (*purple*), template RNA (*black*), nascent RNA (*red*), TRS (*blue*)].***A*, the 5′-UTR coordinates to the nsp13 dimer, with TRS-L bound N protein positioned above the polymerase active site. RNA synthesis begins on the 3′ end of the template. *B*, synthesis continues until the N protein dimerizes with another N protein bound to TRS-B on the template. *C*, the N proteins release the RNA. *D*, the complementary TRS-B of the nascent strand recouples with TRS-L. *E*, the shift in RNA position triggers nsp13 template backtracking, unwinding the dsRNA. *F*, once fully unwound, synthesis continues on the 5′ leader, starting from the TRS-L.

First, the N protein has a general affinity for the TRS sequence (TRS-L or TRS-B), but specificity for TRS-L as the target of the template switch suggests that additional *cis*-acting RNA structural elements play a role. A series of papers on the MHV 5′-UTR structure established that stem loop SL2, which precedes the TRS-L on SL3, is required for transcription (61), while SL4, which immediately follows the TRS-L, also appears to play a role (62). Both elements could provide additional interactions with the nsp13 subunits, helping to secure the N protein bound TRS-L over the polymerase active site. Furthermore, a number of studies have established a long-range interaction between the 5′-UTR and 3′-UTR (63, 64), which implies that the 5′-UTR would be positioned near the polymerase active site during initiation of negative-strand synthesis. In the case of MHV, this genome cyclization occurs with the 5′-UTR SL1 element (63), but with SARS-CoV-2 it appears to occur with SL3 (containing the TRS-L) (64). Indeed, the SL3 sequence (60–80) and the 3′ sequence immediately preceding the poly-A tail (29,847–29,868) are highly complementary. Unwinding of these paired 5′ and 3′ sequences would be a necessary condition for polymerization to start, something the N protein has been demonstrated to do specifically in the case of the TRS sequence (65). All of this is consistent with a picture in which initiation of polymerization on the 3′ end of the genome serves to position the N protein bound TRS-L over the polymerase active site (Fig. 7*A*).

Second, the TRS-L is effectively sequestered by the N protein and would need to be released in order to base pair with the nascent strand. The N protein CTD is known to be a dimerization domain (66). This raises the possibility that an N protein bound to a TRS-B would dimerize with the N protein bound to the TRS-L once that segment of the template reaches the polymerase (Fig. 7*B*). This dimerization could then force the release of the TRS-L (Fig. 7*C*), freeing it up for coupling to the nascent strand once the complementary sequence has been synthesized (Fig. 7*D*). The dynamics of this process are likely complicated and have not yet been investigated. It is unclear if this would be a stepwise process or more concerted. But the elements of proximity and the ability of the N protein to both recognize the TRS and dimerize in principle should be sufficient to facilitate a recoupling of the nascent strand from TRS-B to TRS-L.

Finally, the original template would need to unwind from the nascent strand for polymerization to continue on the new template. As with proofreading, we propose the shift in the dsRNA position upon base pairing of the nascent strand to the TRS-L triggers activation of nsp13 and backtracking of the template. This would unwind the original template from the nascent strand, while leaving it base paired with the new template (Fig. 7*E*). Once unwinding is complete, the dsRNA formed at the TRS-L juncture with the new template could shift back into the polymerase active site and finish synthesis on the remaining nucleotides of the 5′ leader (Fig. 7*F*). This critical role of nsp13 in transcription is supported by a study on the avian infectious bronchitis virus, in which a single mutation introduced to nsp13 (R132P) significantly attenuated the formation of subgenomic transcripts, but had no impact on full-genome synthesis (67).

We should note that binding of the N protein to the nsp13 dimer appears to block access to the nsp13.1 RNA-binding groove, suggesting that nsp13.2 may govern backtracking in this situation. While the template has so far been seen by cryo-EM only engaging with nsp13.1, modeling of the relevant states indicates that nsp13.2 could perform this backtracking function as well. Thus, it may be that nsp13.1 governs backtracking for proofreading during positive-strand synthesis, but nsp13.2 governs backtracking for proofreading and transcription during negative-strand synthesis.

### Summary

The detailed atomistic model of the SARS-CoV-2 RTC was derived in part from a series of protein–protein docking exercises. It offers a detailed protein–protein interaction map in which hexameric nsp15 forms a larger complex with nsp14/nsp10 and nsp16/nsp10, which is then capable of recruiting the polymerase complex. An analysis of the structure leads to a consistent picture of RNA processing, offering new hypotheses on the functional roles of its components. Several key hypotheses include the following:

1) Nsp13 functions more as a translocase than a helicase, facilitating backtracking of the template strand during proofreading and template switching. It is proposed to be activated when the dsRNA is sensed to be out of position in the polymerase, likely *via* nsp8.
2) Nsp14 ExoN sits on the dsRNA exit side of nsp12. It is proposed to engage in proofreading when the prematurely 3′ terminated dsRNA is translocated to the site following a mismatch incorporation. The RTC is reset to polymerization *via* backtracking when nsp13 acts on the template strand.
3) Nsp15 EndoN is proposed to act on the template strand, likely near the 5′ terminus. Its preference for uridine suggests it acts on the 5′ tail of the negative strand once positive-strand synthesis is complete.
4) The N protein bound to the TRS oligo coordinates to the two nsp13 subunits, positioning the RNA over the polymerase active site, with clear implications for template switching. Nsp13 is proposed to unwind the original template from the nascent strand following the template switch.
5) Multiple zinc fingers from nsp14 and nsp10 converge over the dsRNA after passing over the EndoN site to facilitate strand separation.
6) Nsp12 NiRAN is proposed to transfer GDP to pppN-RNA with a loss of pyrophosphate in the first step of mRNA capping.

While further simulations are necessary, as is experimental confirmation, the model offers a framework for interpreting a range of observations, serving as a guide for future assay development, mechanistic and structural studies, and investigations of new drug targets.

## Experimental procedures

Structures used to construct the model were the following: nsp12/nsp7/nsp8/nsp13/dsRNA (PDB:6XEZ) (32); nsp15 (PDB:6X1B) (25); nsp16/nsp10 (PDB:6WVN) (21); nsp9 (PDB:6W4B) (K. Tan, unpublished results); and N NTD/TRS-L (PDB:7ACT) (31). Since an X-ray structure of nsp14 was not available at the time this work was done, an homology model was built based on the SARS structure (PDB:5NFY, chains A/M) (6).

Protein–protein docking was carried out using the Piper (68) method within Bioluminate (69). The program was run with default settings, in which 70,000 orientations of the “ligand” protein with respect to the “receptor” protein were sampled and scored. The top 30 poses were returned, which we then sorted into clusters. In some cases, as indicated in the text, the top scoring pose appeared to carry functional significance and was chosen for refinement. In other cases, refinement was done on multiple poses before selecting the best candidate for further work. No pose outside of the top ten was ever chosen for follow-up. As noted in the text, the only exceptions to this procedure were the placement of nsp14-ExoN/nsp10.14 on the nsp15 hexamer and the placement of nsp12 on the core complex, both of which were positioned manually, guided by aligning bound dsRNA sections. The RNA was subsequently fused to facilitate optimization.

Refinement was an iterative process within the Schrödinger suite that involved sidechain and loop conformation optimization through Prime (70), minimization through Prime and Macromodel (71), and additional conformational sampling through Macromodel. The forcefield employed was OPLS4 (72). In each case, optimization was initially limited to the residues that form the protein–protein interface of interest and was generally carried out with Prime. Once this step was completed, the “ligand” protein was allowed to fully relax. Once the initial model was constructed, all proteins were replaced with their original PDB structures and reoptimized. Missing residues, primarily in the N-term and C-term tails, were added where necessary.

The section of dsRNA that extends from the Pol active site to the EndoN site was first minimized in Macromodel using tight constraints to maintain Watson–Crick base pairing. These constraints were reduced in a second round of optimization. Particular attention was given to the RNA at the site of unwinding and along the path of the nascent strand toward the NiRAN site. This was done by extending the RNA 3 to 4 nucleotides at a time and running a Macromodel conformational search of up to six nucleotides at a time. Basic residues in contact with the RNA were also sampled in these conformational searches.

The final structure was minimized in sections, due to practical limits in dealing with such a large complex.

## Data availability

The model described here is available in PDB format as part of the Supplementary Information.

## Supporting information

This article contains supporting information.

## Conflict of interest

The authors declare that they have no conflicts of interest with the contents of this article.
