# Supplementary material for: An atomistic model of the coronavirus replication-transcription complex as a hexamer assembled around nsp15
Source: J Biol Chem. 2021 Sep 23;297(4):101218. doi: 10.1016/j.jbc.2021.101218 (PMC8494237; doi:10.1016/j.jbc.2021.101218)
Supplement: Supplemental Figures S1–S5 and Tables S1, S2 [file mmc1.pdf]

Supplementary Material

An atomistic model of the coronavirus replication-transcription complex as a hexamer assembled around nsp15

Jason K. Perry,[1*] Todd C. Appleby,[1] John P. Bilello,[1] Joy Y. Feng,[1] Uli Schmitz,[1] and Elizabeth A. Campbell[2]

[1]Gilead Sciences, Inc., Foster City, CA, 94404

[2]Laboratory of Molecular Biophysics, The Rockefeller University, New York, NY 10065

[*]Correspondence: jason.perry@gilead.com

Table S1. Residues defining the nsp14/nsp15 interface. Residues are within 3 Å of the other protein.

Protein	Residues
nsp14	N62, Q64, N66, R80, E294, A336, I337, K338, P341, Q342, D344, K348, F349, K358, Y360, K361, D448, S449, P450, E452, H485
nsp15	V131, T144, E145, G146, S147, V148, K149, Q152, P153, S154, V155, Q175, Q188, L189, P190, E191, T192, Y193, K226, V313, V314, K316, V317, V318, K319, V320, Y324, E326, K334

Table S2. Residues defining the nsp12/nsp14 interface. Residues are within 3 Å of the other protein.

Protein	Residues
nsp12	R583, Y595, G596, H599, N600, K603, V820, K821, Q822, G823, D824, V827, L829, I864, Y867, T870, K871, A878, F881, H882, L885, Q886, R889, D910, S913, R914, W916, E917, P918, E919, Y921, E922, Y925, P927, H928, T929, L931
nsp14	D143, Q144, K146, N251, Q253, S254, D257, L258, Y259, Q261, V262, H263, G264, N265, A266, H267, D389, R390, R399, R403, L405, S406, L410, P411, G412, C413, D414, G415, G416, L418, V420, H423, A424, H426, P428, A429, F430, D431, K432, S433

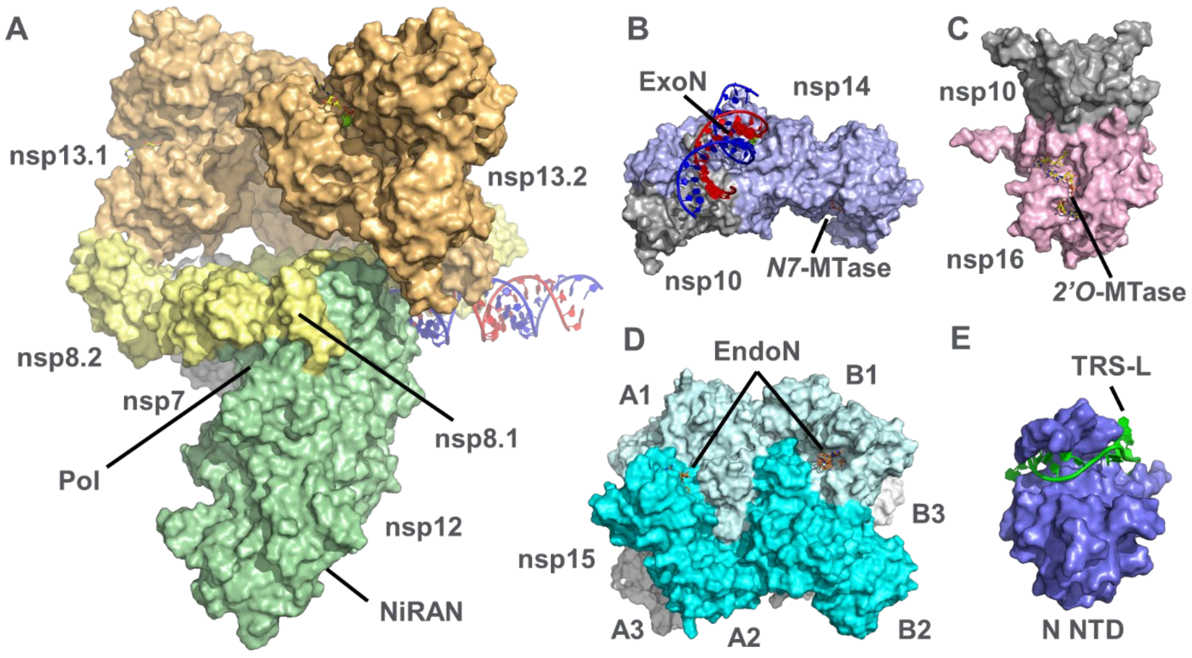

Figure S1. Key components of the coronavirus RTC. *A,* SARS-CoV-2 polymerase complex of nsp12 (green), nsp13 (orange), nsp7 (white) and nsp8 (yellow) (PDB: 6XEZ). *B,* Homology model of SARS-CoV-2 ExoN/*N7*-MTase nsp14 (blue) with nsp10 (gray) cofactor (based on SARS-CoV PDB: 5NFY, with dsRNA modeled after Lassa ExoN PDB: 4FVU). *C,* SARS-CoV-2 *2'O*-MTase nsp16 (pink) with nsp10 cofactor (PDB: 6WVN). *D,* Hexamer of SARS-CoV-2 EndoN nsp15 (cyan) (PDB: 6X1B). Subunits of the nsp15 hexamer are labeled, reflecting two trigonal faces (A1/A2/A3 and B1/B2/B3). *E,* SARS-CoV-2 N protein NTP (residues 44-180) bound to the 10 nt TRS-L oligo (PDB: 7ACT).

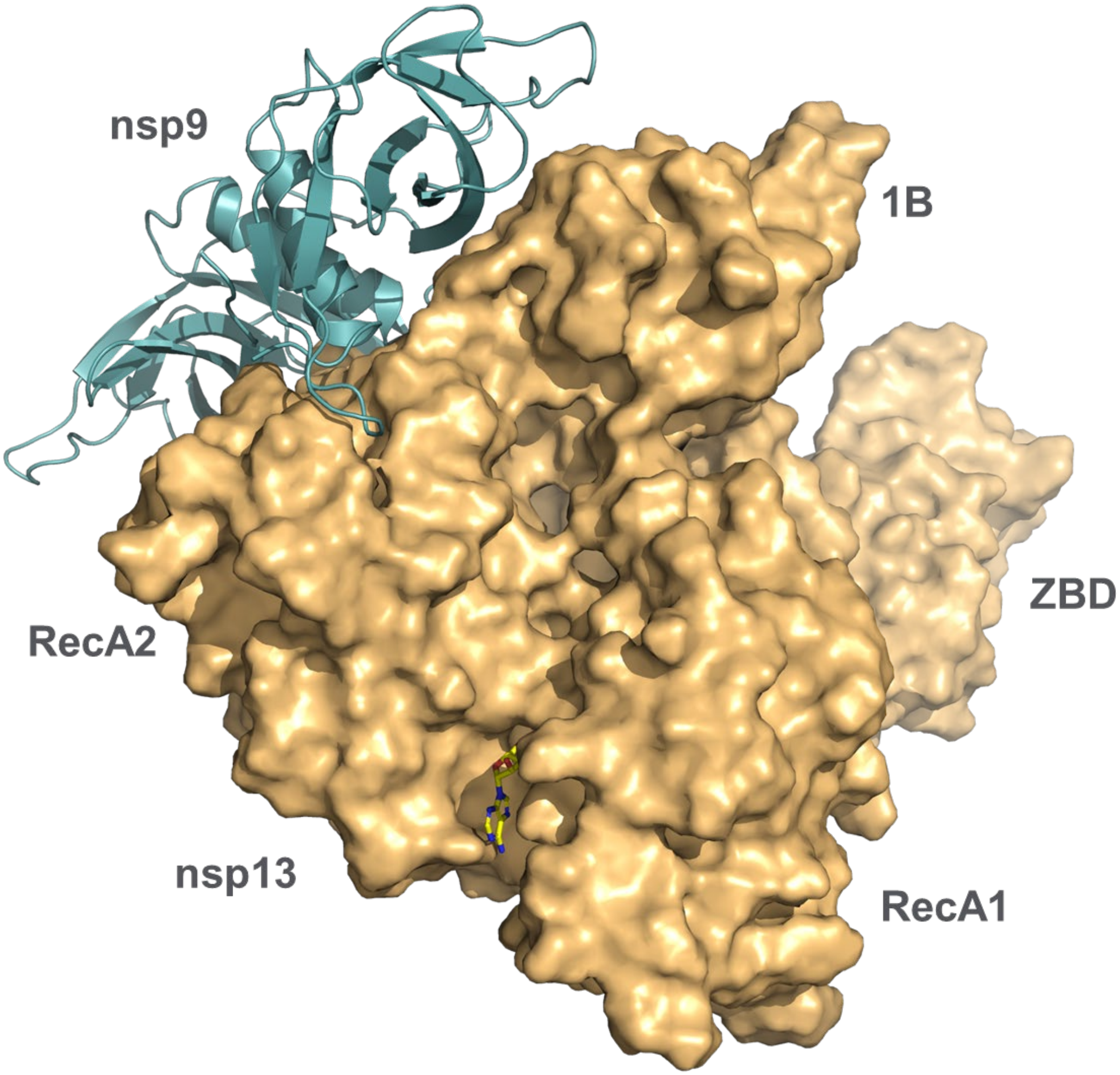

Figure S2. Docking of the nsp9 dimer to the active state of nsp13. Nsp9 coordinates to the RecA2 domain, where it is positioned to interact with the 5’ end of RNA as it exits the nsp13 RNA binding channel. A similar binding mode was found for other conformations of nsp13.

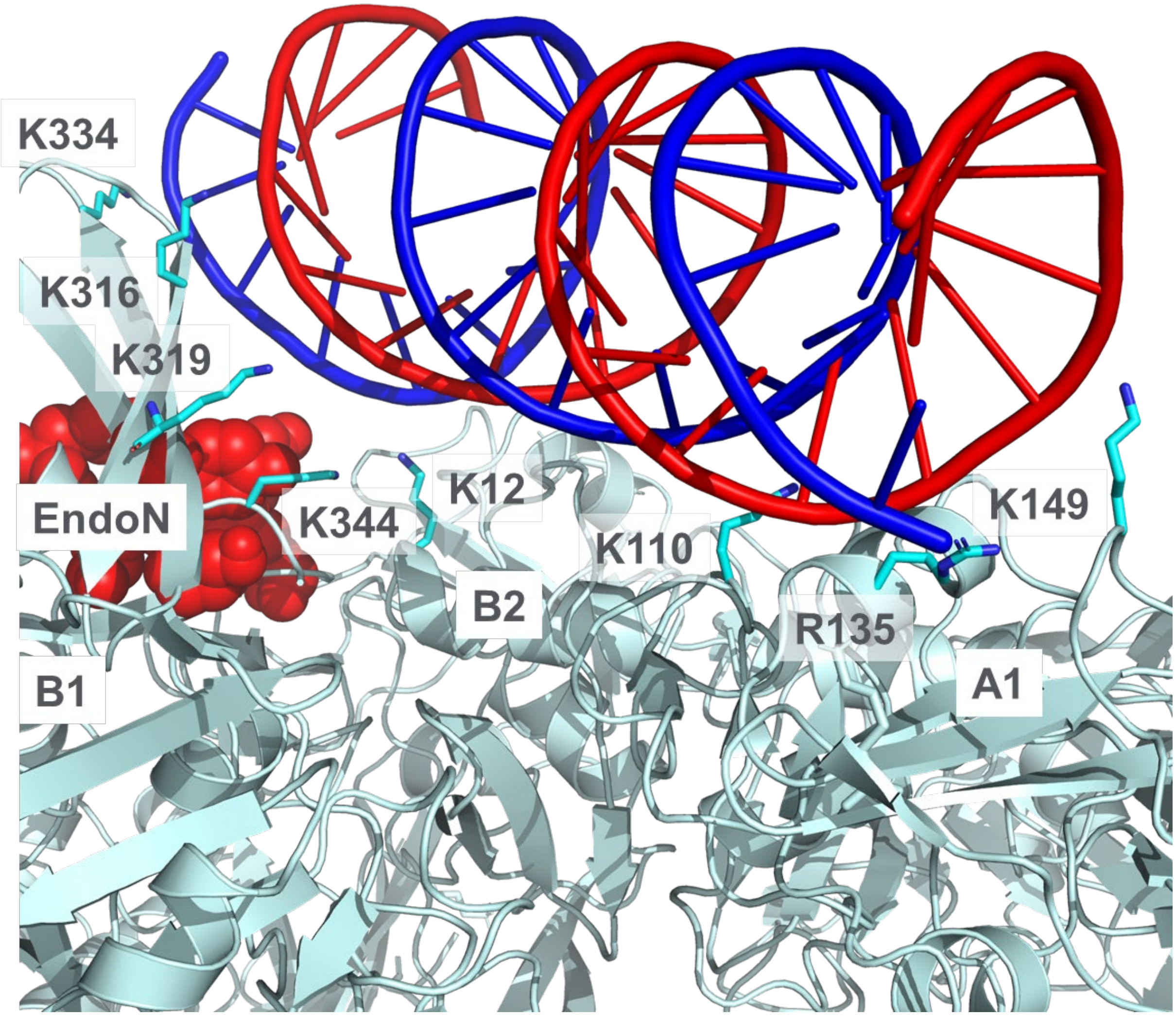

Figure S3. Detail of dsRNA path over hexameric nsp15. The RNA interacts with eight basic residues from three different subunits and passes over the EndoN site (red).

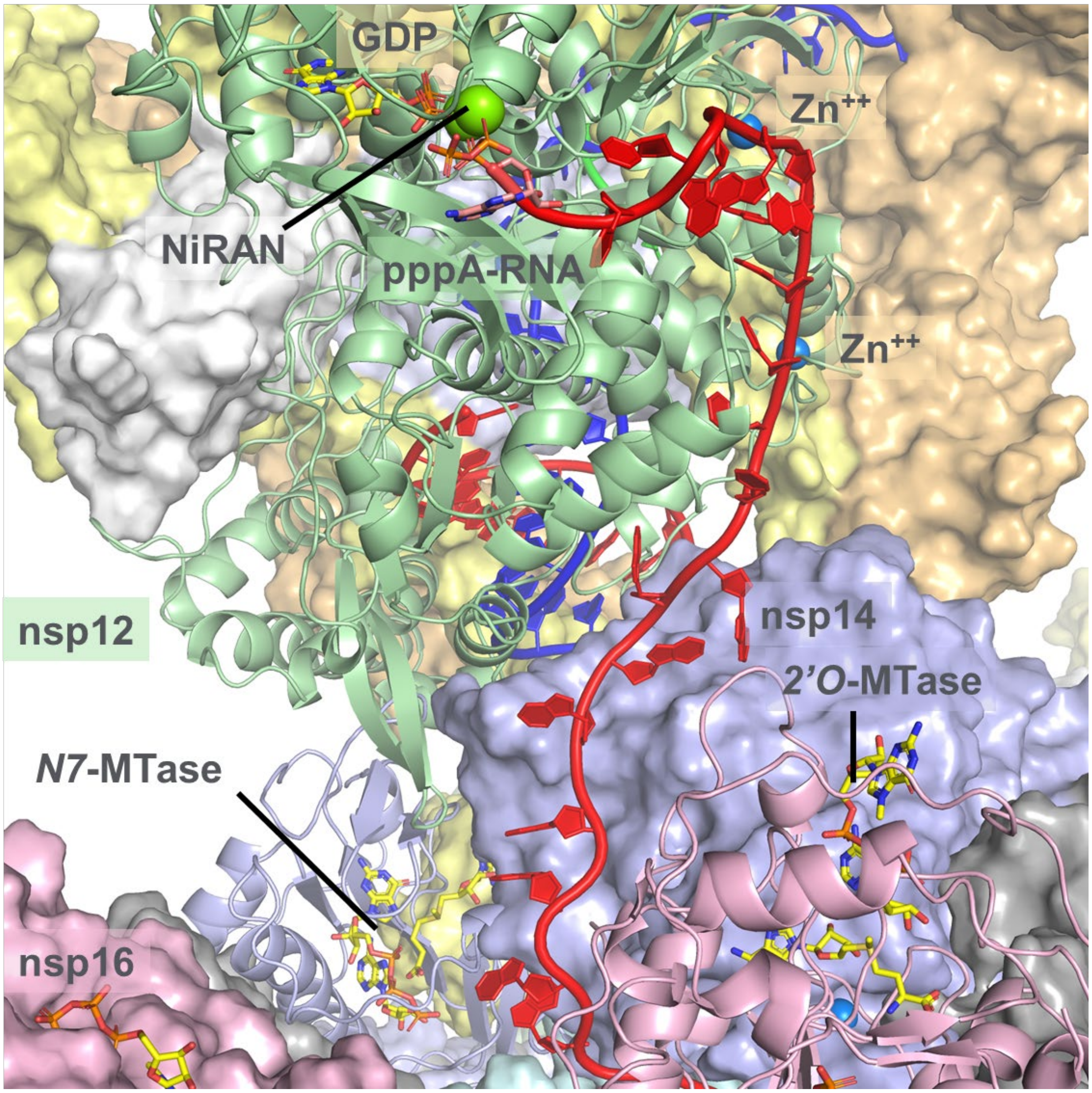

Figure S4. Detail of the nascent strand RNA emerging from a basic residue rich channel after strand separation and making its way to the NiRAN domain to initiate capping. The RNA passes by several zinc fingers, including two on nsp12. After the initial transfer of G to the pppA-RNA, the RNA makes its way to the nsp14 *N7*-MTase and nsp16 *2'O*-MTase to complete the mRNA capping.

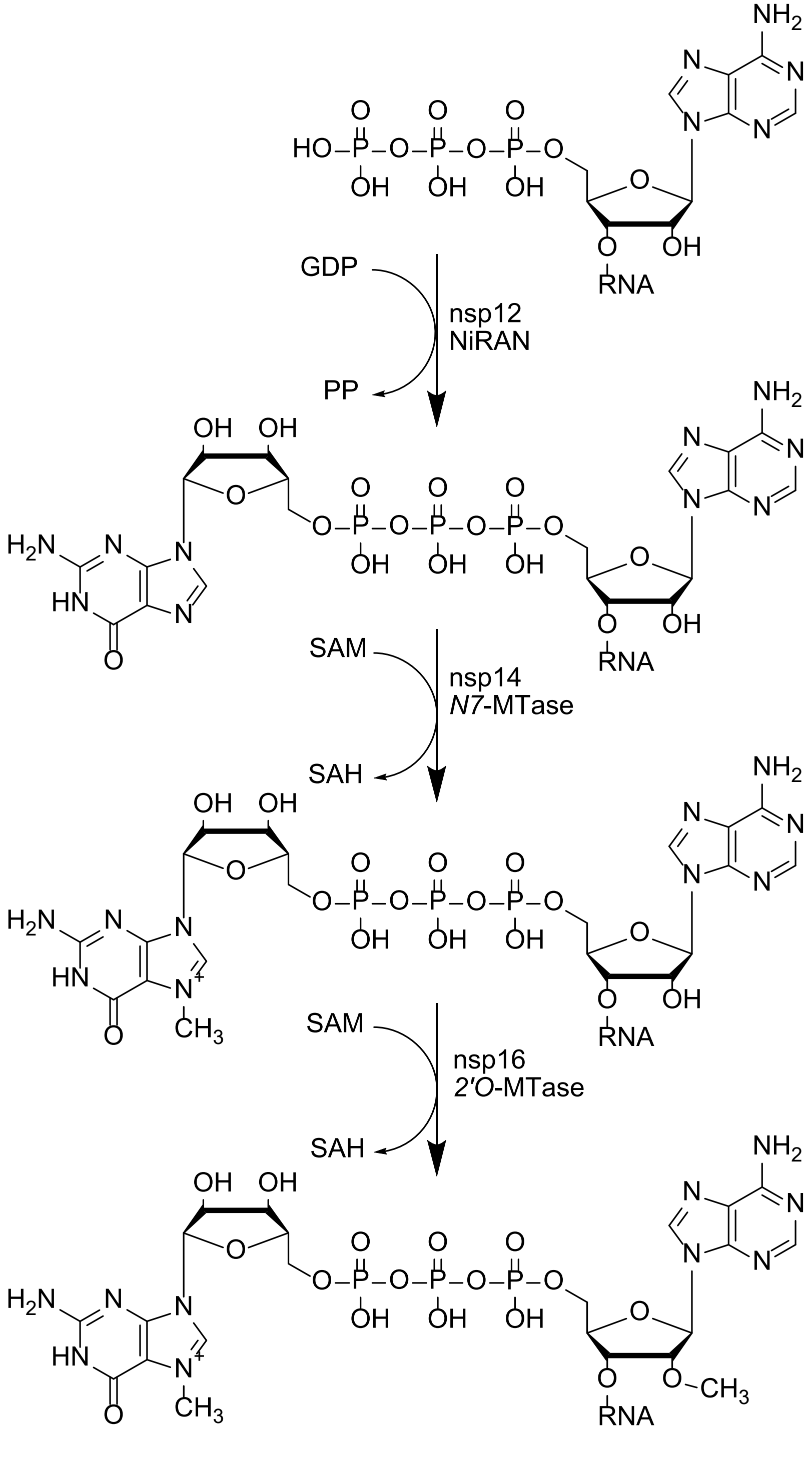

Figure S5. Proposed mRNA capping schematic. The nsp12 NiRAN site facilitates transfer of GDP to the 5’ pppA-RNA, with loss of pyrophosphate. The *N7* of the G is then methylated via the nsp14 MTase. Finally, the *2’O* of the A is methylated via the nsp16 MTase.
